# Supplementary material for: Unraveling the role of Ctla-4 in intestinal immune homeostasis through a novel Zebrafish model of inflammatory bowel disease
Source: eLife. 2025 May 20;13:RP101932. doi: 10.7554/eLife.101932 (PMC12092003; doi:10.7554/eLife.101932)
Supplement: Figure 1—source data 1. [file elife-101932-fig1-data1.zip › Figure 1-Source Data 1.pdf]

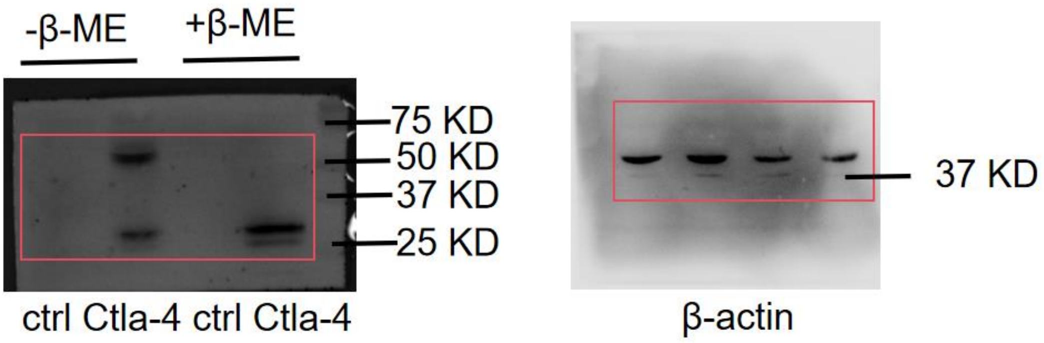

Figure 1-Source Data 1. PDF file containing original western blots for Figure 1C, indicating the relevant bands and treatments.
