# Supplementary figures and images for: Unraveling the role of Ctla-4 in intestinal immune homeostasis through a novel Zebrafish model of inflammatory bowel disease
Source: eLife. 2025 May 20;13:RP101932. doi: 10.7554/eLife.101932 (PMC12092003; doi:10.7554/eLife.101932)

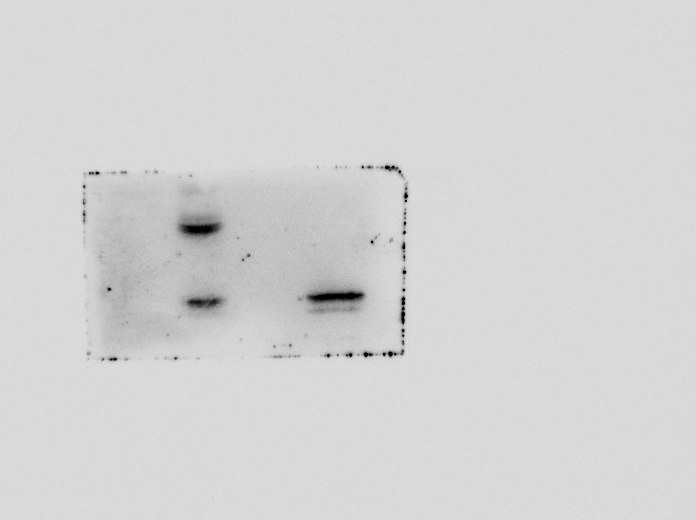

Supplement: Figure 1—source data 2. [file elife-101932-fig1-data2.zip › Figure 1-Source Data 2/Original files for western blot analysis displayed in Figure 1C-Ctla-4.tif]

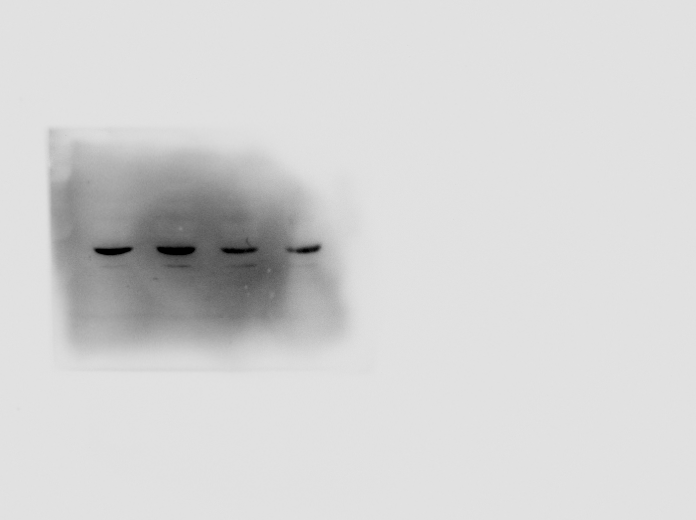

Supplement: Figure 1—source data 2. [file elife-101932-fig1-data2.zip › Figure 1-Source Data 2/Original files for western blot analysis displayed in Figure 1C-β-actin.tif]

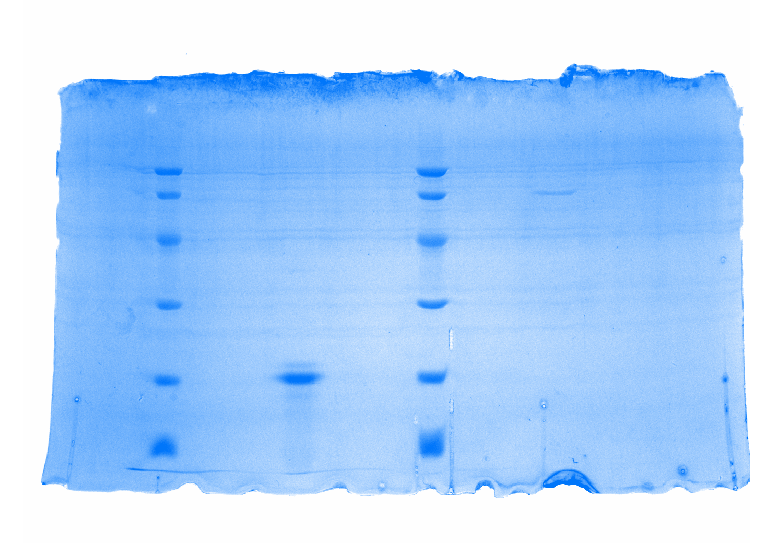

Supplement: Figure 1—figure supplement 2—source data 2. [file elife-101932-fig1-figsupp2-data2.zip › Original files displayed in Figure 1-figure supplement 2A-the protein of Ctla-4-ECD.tif]

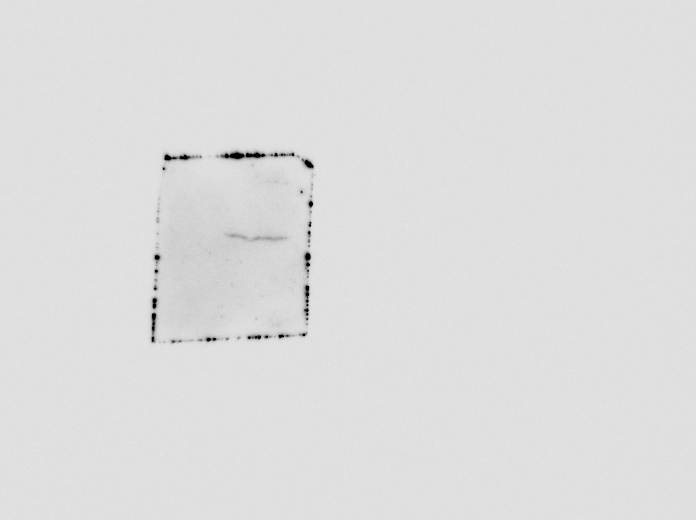

Supplement: Figure 1—figure supplement 2—source data 2. [file elife-101932-fig1-figsupp2-data2.zip › Original files displayed in Figure 1-figure supplement 2B-the Ctla-4 was tested in zebrafish intestines.tif]

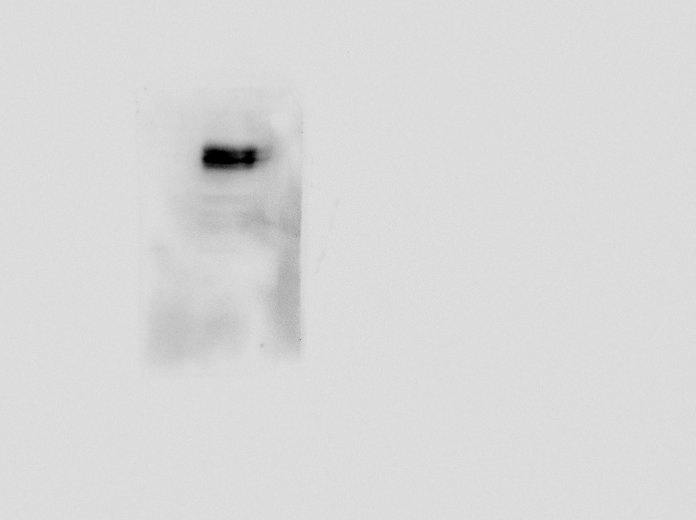

Supplement: Figure 1—figure supplement 2—source data 2. [file elife-101932-fig1-figsupp2-data2.zip › Original files displayed in Figure 1-figure supplement 2B-the Ctla-4-EGFP was tested by anti-Ctla-4.tif]

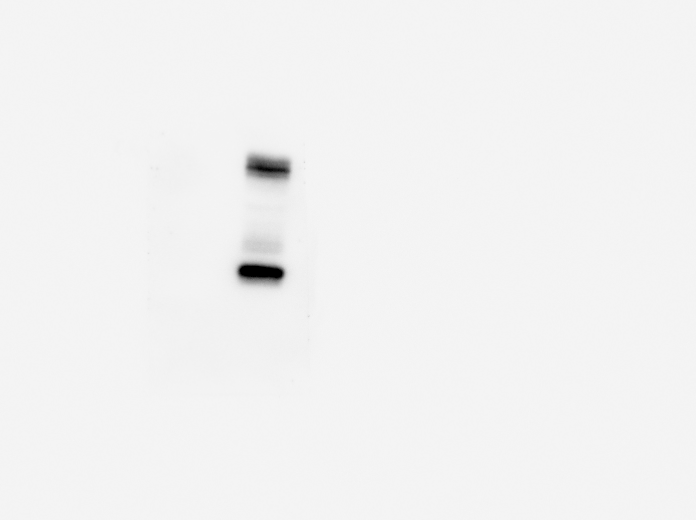

Supplement: Figure 1—figure supplement 2—source data 2. [file elife-101932-fig1-figsupp2-data2.zip › Original files displayed in Figure 1-figure supplement 2B-the Ctla-4-EGFP was tested by anti-EGFP.tif]

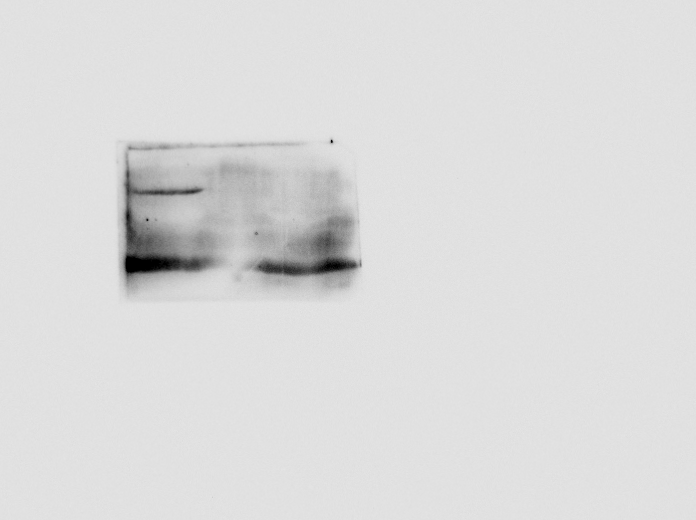

Supplement: Figure 2—source data 2. [file elife-101932-fig2-data2.zip › Figure 2-Source Data 2/Original files for western blot analysis displayed in Figure 2C-The Ctla-4 in intestine.tif]

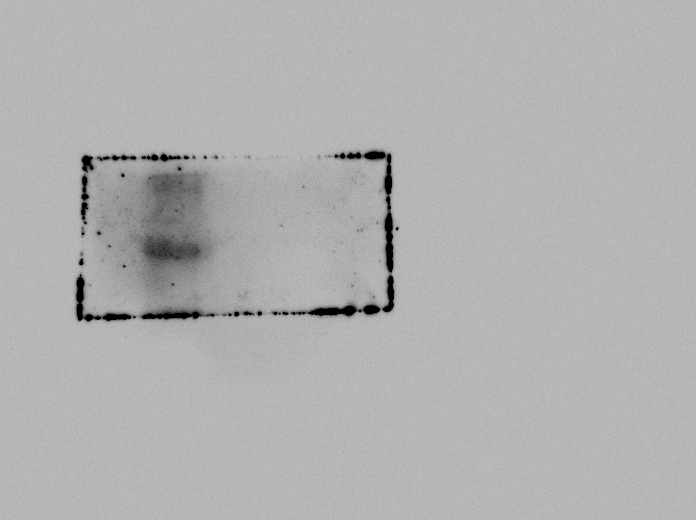

Supplement: Figure 2—source data 2. [file elife-101932-fig2-data2.zip › Figure 2-Source Data 2/Original files for western blot analysis displayed in Figure 2C-The Ctla-4 in spleen.tif]

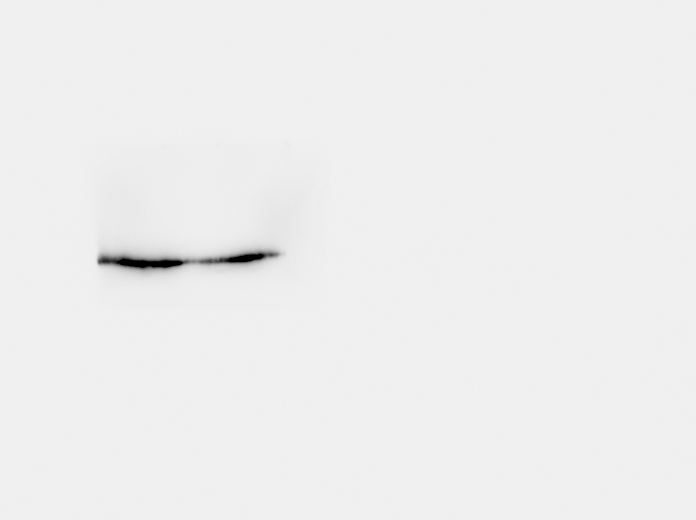

Supplement: Figure 2—source data 2. [file elife-101932-fig2-data2.zip › Figure 2-Source Data 2/Original files for western blot analysis displayed in Figure 2C-The Gapdh in intestine.tif]

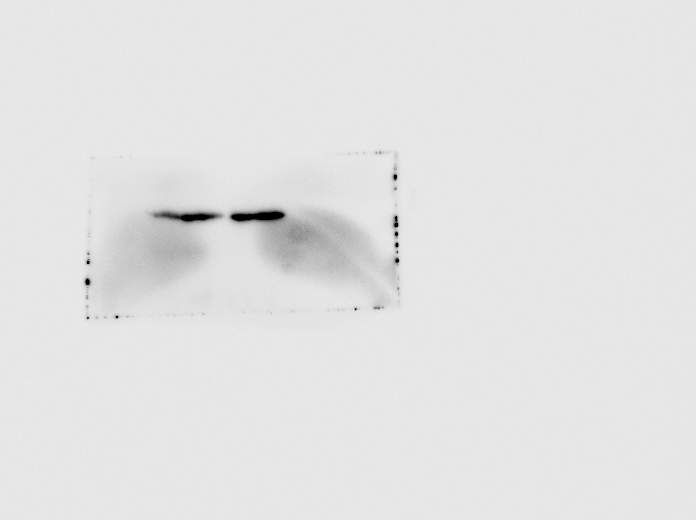

Supplement: Figure 2—source data 2. [file elife-101932-fig2-data2.zip › Figure 2-Source Data 2/Original files for western blot analysis displayed in Figure 2C-The Gapdh in spleen.tif]

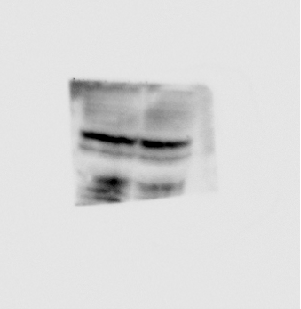

Supplement: Figure 7—source data 2. [file elife-101932-fig7-data2.zip › Figure 7-Source Data 2/Original files for western blot analysis displayed in Figure 7H-Cd28-input-the interaction between Cd28 and Cd86.tif]

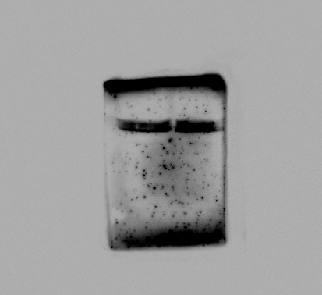

Supplement: Figure 7—source data 2. [file elife-101932-fig7-data2.zip › Figure 7-Source Data 2/Original files for western blot analysis displayed in Figure 7H-Cd28-IP-the interaction between Cd28 and Cd86.tif]

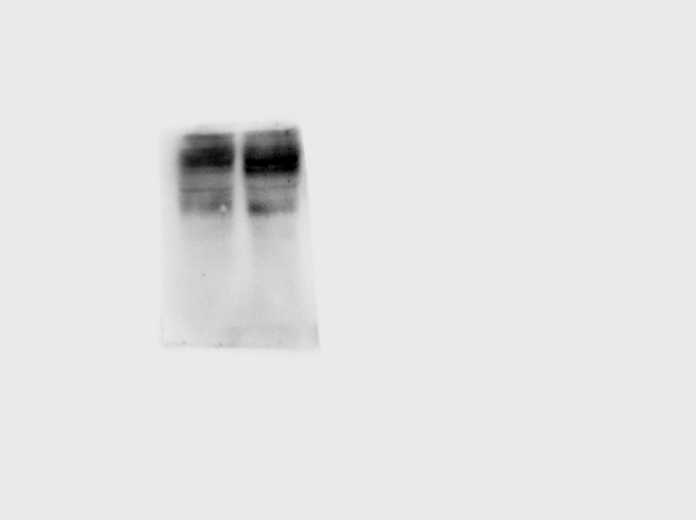

Supplement: Figure 7—source data 2. [file elife-101932-fig7-data2.zip › Figure 7-Source Data 2/Original files for western blot analysis displayed in Figure 7H-Cd86-input-the interaction between Ctla-4 and Cd86.tif]

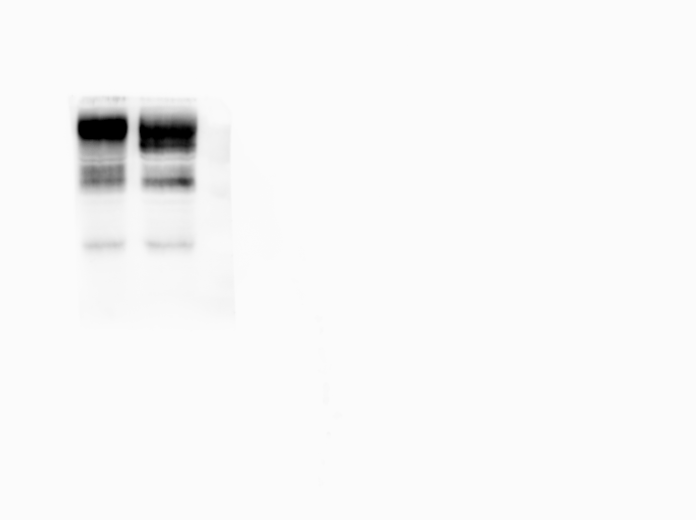

Supplement: Figure 7—source data 2. [file elife-101932-fig7-data2.zip › Figure 7-Source Data 2/Original files for western blot analysis displayed in Figure 7H-Cd86-IP-the interaction between Ctla-4 and Cd86.tif]

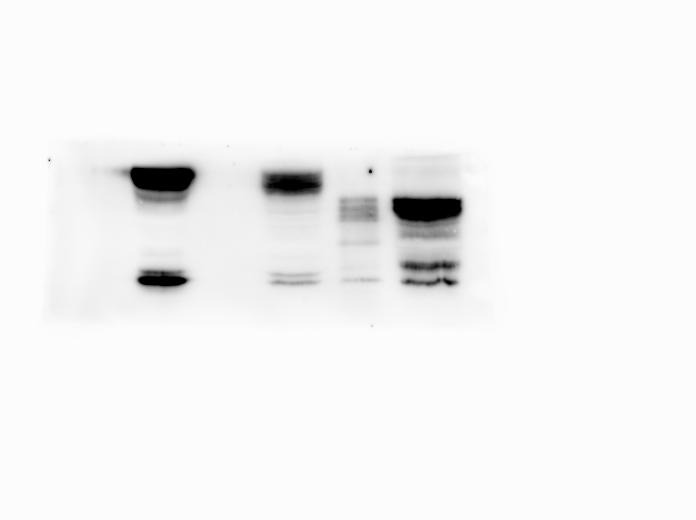

Supplement: Figure 7—source data 2. [file elife-101932-fig7-data2.zip › Figure 7-Source Data 2/Original files for western blot analysis displayed in Figure 7H-Cd86-the interaction between Cd28 and Cd86.tif]

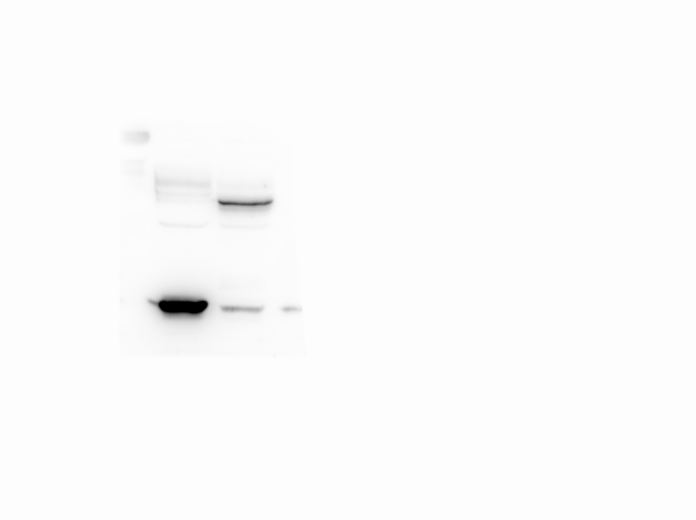

Supplement: Figure 7—source data 2. [file elife-101932-fig7-data2.zip › Figure 7-Source Data 2/Original files for western blot analysis displayed in Figure 7H-Ctla-4-input-the interaction between Ctla-4 and Cd86.tif]

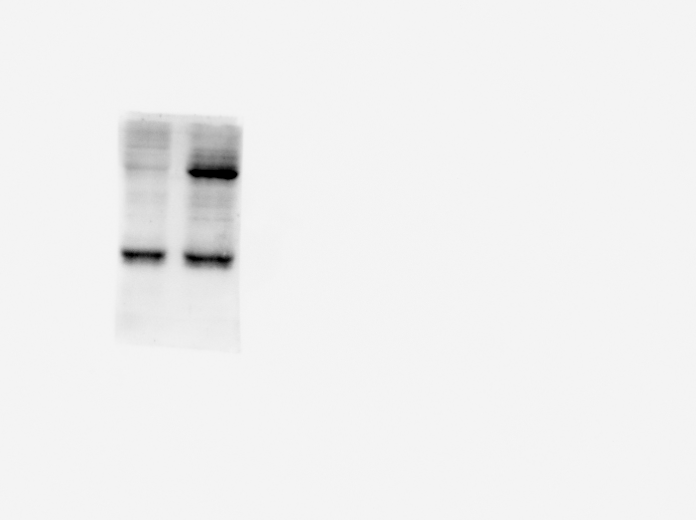

Supplement: Figure 7—source data 2. [file elife-101932-fig7-data2.zip › Figure 7-Source Data 2/Original files for western blot analysis displayed in Figure 7H-Ctla-4-IP-the interaction between Ctla-4 and Cd86.tif]

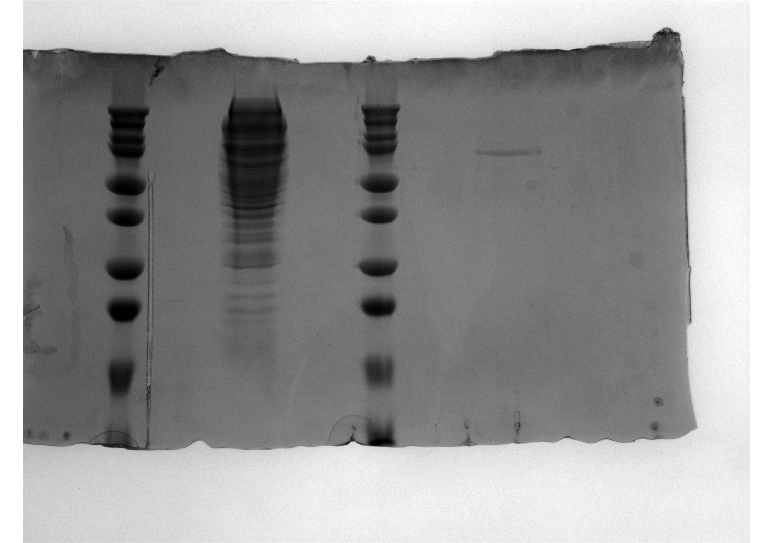

Supplement: Figure 7—figure supplement 1—source data 2. [file elife-101932-fig7-figsupp1-data2.zip › Original files displayed in Figure 7-figure supplement 1A-the Ctla-4-Ig.tif]

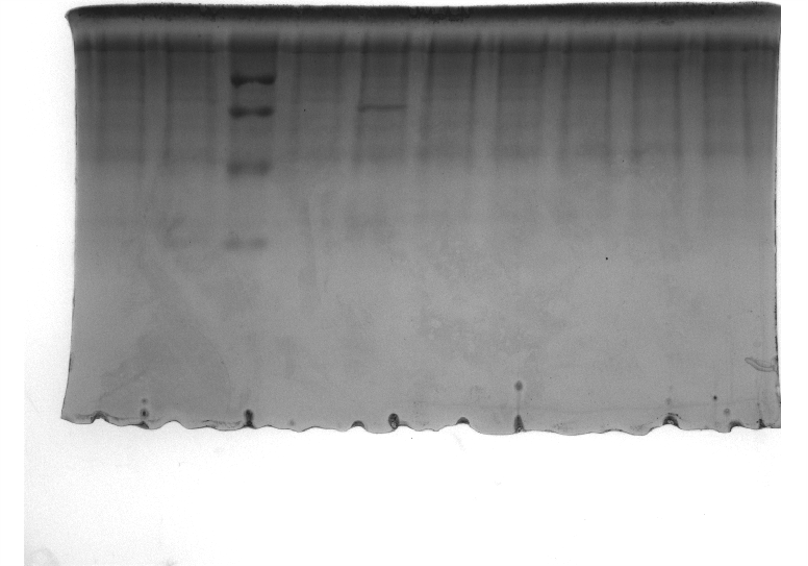

Supplement: Figure 7—figure supplement 1—source data 2. [file elife-101932-fig7-figsupp1-data2.zip › Original files displayed in Figure 7-figure supplement 1B-the Cd28-Ig.tif]

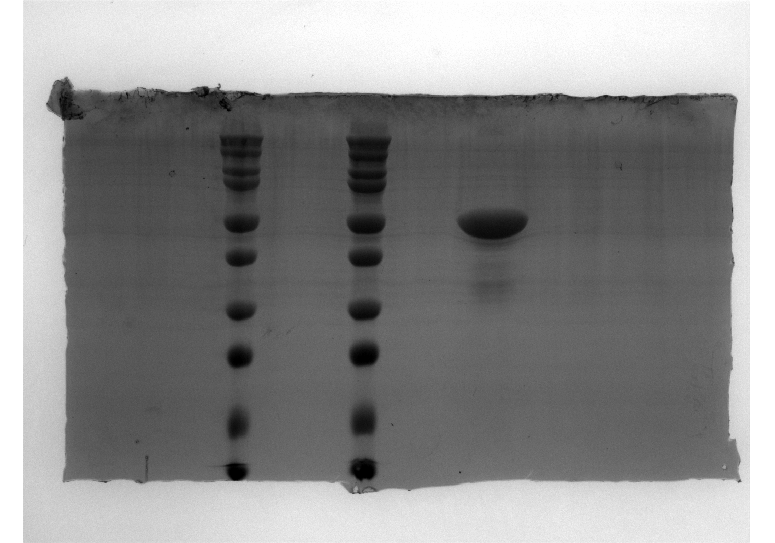

Supplement: Figure 7—figure supplement 1—source data 2. [file elife-101932-fig7-figsupp1-data2.zip › Original files displayed in Figure 7-figure supplement 1C-the Cd86.tif]
