# Supplementary material for: Unraveling the role of Ctla-4 in intestinal immune homeostasis through a novel Zebrafish model of inflammatory bowel disease
Source: eLife. 2025 May 20;13:RP101932. doi: 10.7554/eLife.101932 (PMC12092003; doi:10.7554/eLife.101932)
Supplement: Figure 1—figure supplement 2—source data 1. [file elife-101932-fig1-figsupp2-data1.pdf]

A

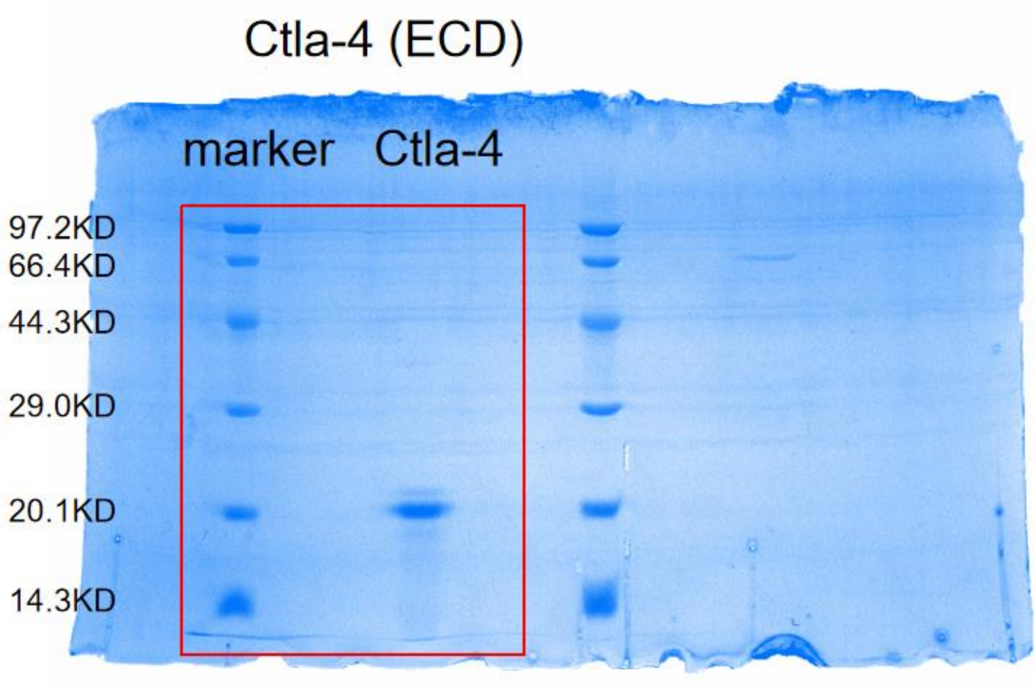
Ctla-4 (ECD)
marker
Ctla-4
97.2KD
66.4KD
44.3KD
29.0KD
20.1KD
14.3KD

B

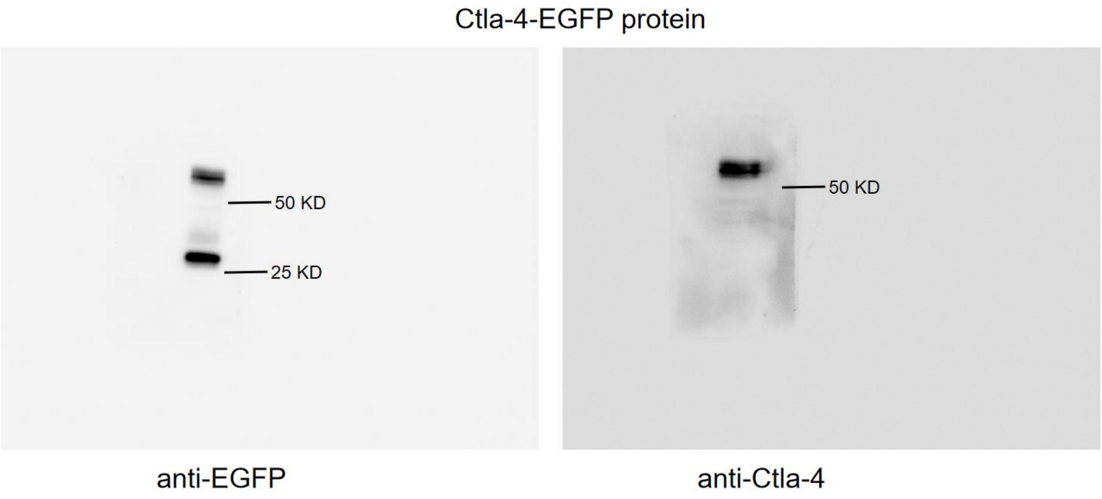
Ctla-4-EGFP protein
50 KD
25 KD
50 KD
anti-EGFP
anti-Ctla-4

C

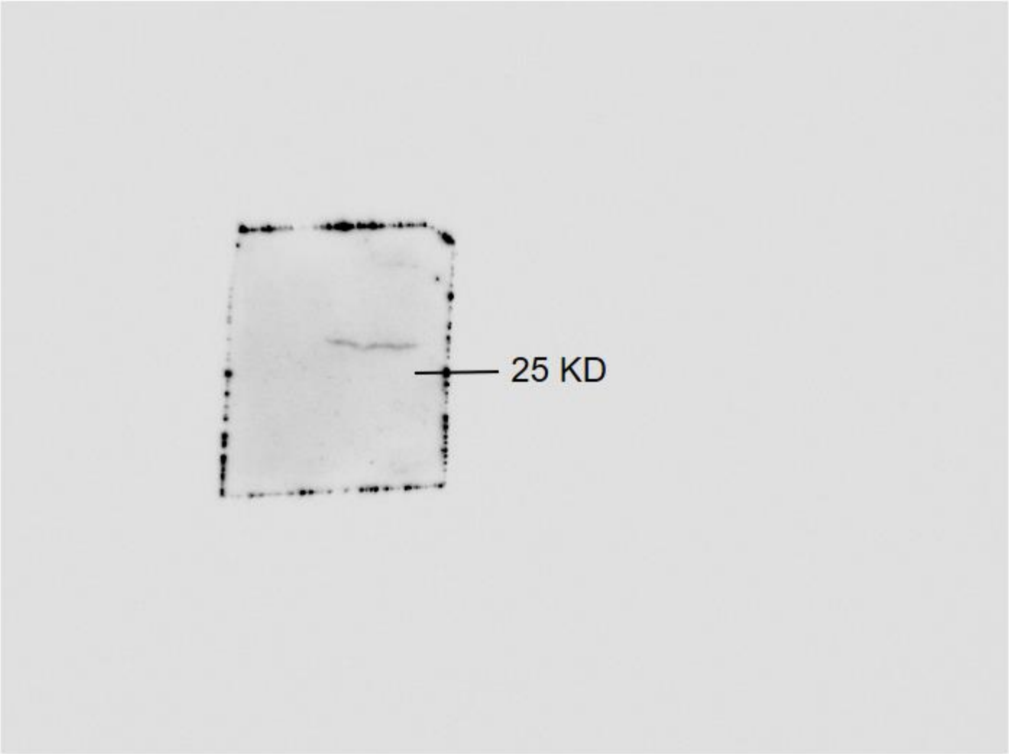

Figure 1-figure supplement 2-Source Data 1. PDF file containing original western blots for Figure 1-figure supplement 2A-C. A SDS-PAGE detection of the recombinant Cytotoxic T lymphocyte antigen-4 (Ctla-4) protein with extracellular domain (ECD). B Western blot analysis of the mouse anti-EGFP and anti-Ctla-4 antibodies that bind to the recombinant Ctla-4-EGFP fusion proteins expressed in HEK293T cells. C Western blot analysis of native Ctla-4 protein in zebrafish intestinal tissues using mouse anti-Ctla-4 antibody.
