# Supplementary material for: Unraveling the role of Ctla-4 in intestinal immune homeostasis through a novel Zebrafish model of inflammatory bowel disease
Source: eLife. 2025 May 20;13:RP101932. doi: 10.7554/eLife.101932 (PMC12092003; doi:10.7554/eLife.101932)
Supplement: Figure 7—source data 1. [file elife-101932-fig7-data1.pdf]

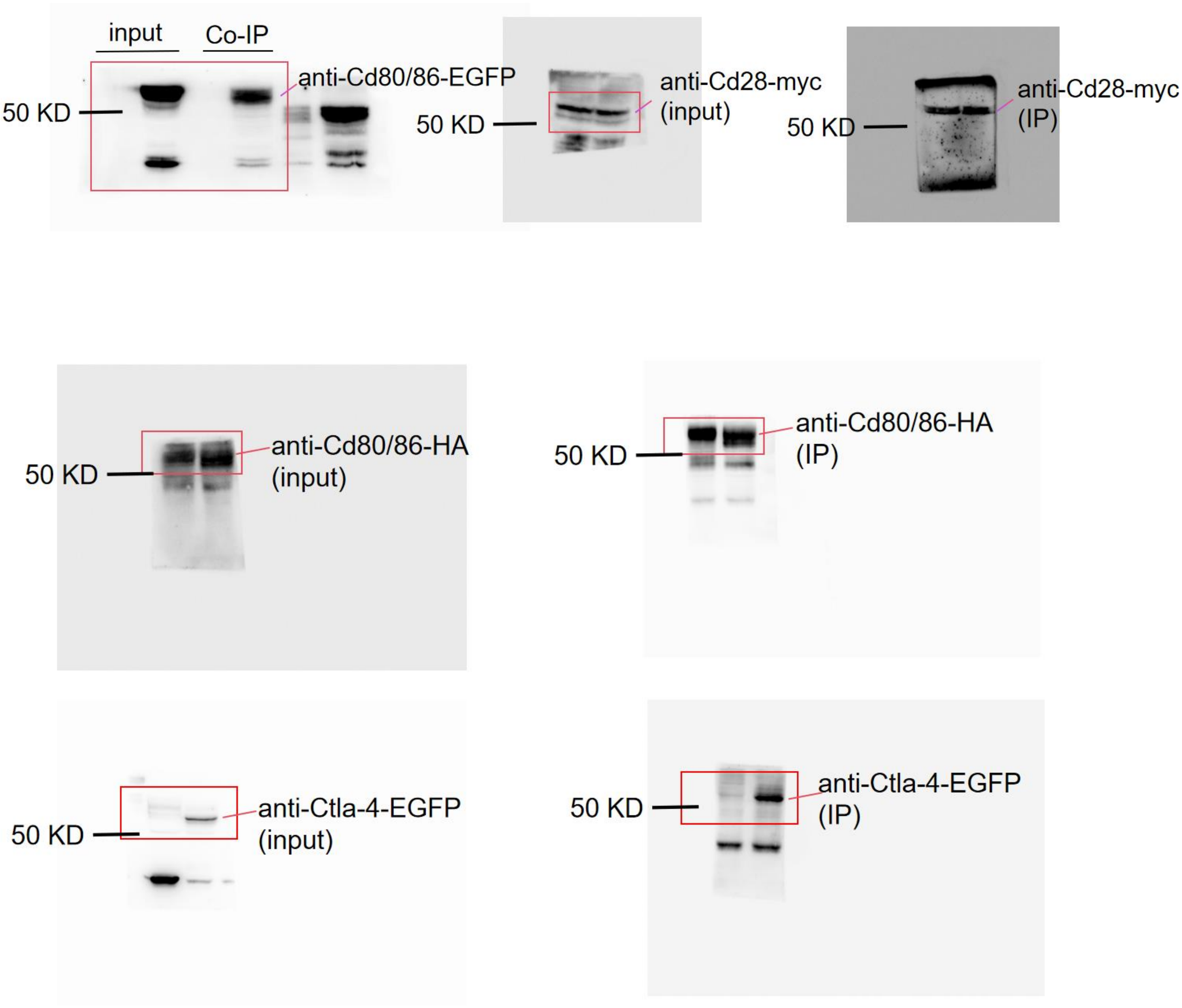

Figure 7-Source Data 1. PDF file containing original western blots for Figure 7H, indicating the relevant bands. The interaction between Cd80/86 and Cd28 (up), and Cd80/86 and Ctla-4 (down) were verified by Co-IP.
