# Supplementary material for: Unraveling the role of Ctla-4 in intestinal immune homeostasis through a novel Zebrafish model of inflammatory bowel disease
Source: eLife. 2025 May 20;13:RP101932. doi: 10.7554/eLife.101932 (PMC12092003; doi:10.7554/eLife.101932)
Supplement: Figure 7—figure supplement 1—source data 1. [file elife-101932-fig7-figsupp1-data1.pdf]

A

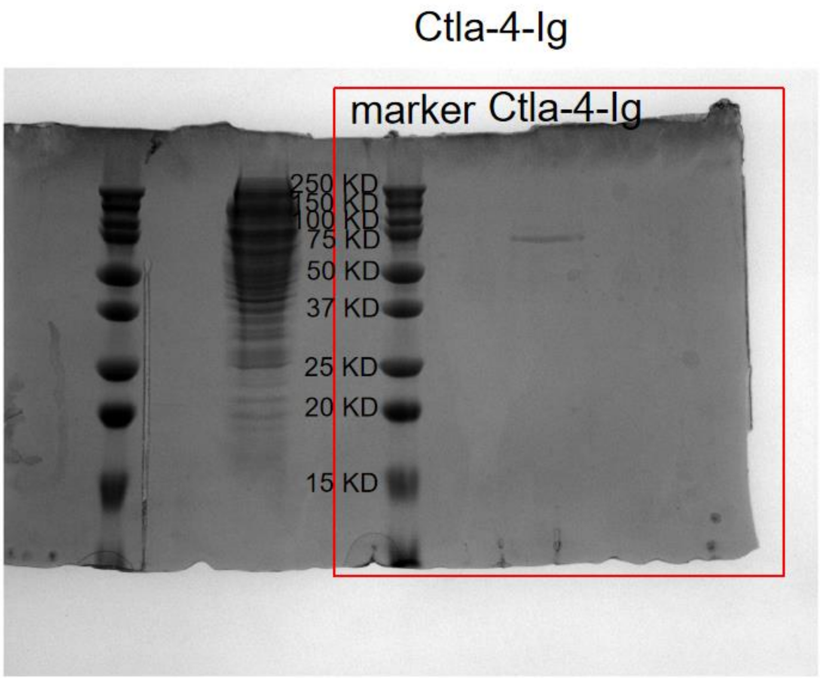
Ctla-4-Ig
marker Ctla-4-Ig
250 KD
150 KD
100 KD
75 KD
50 KD
37 KD
25 KD
20 KD
15 KD

B

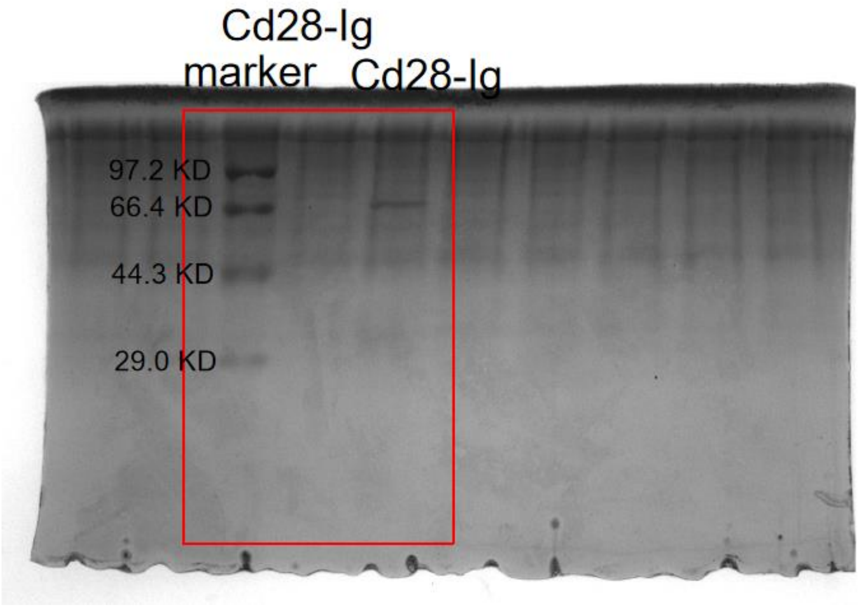
Cd28-Ig
marker Cd28-Ig
97.2 KD
66.4 KD
44.3 KD
29.0 KD

C

Cd80/86 (ECD)

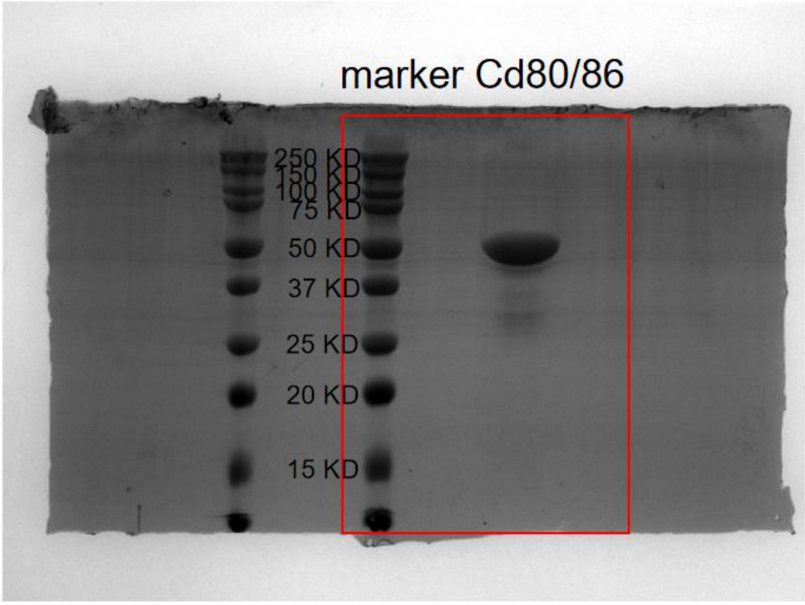


**Figure 7-figure supplement 1-Source Data 1.** PDF file containing original western blots for Figure 7-figure supplement 1A-C. SDS-PAGE detection of the purified recombinant soluble Ctla-4-Ig (sCtla-4) (A) and sCd28-Ig (B) proteins and the Cd80/86 extracellular domain (ECD) (C) with Coomassie brilliant blue staining.
